# Association between Weekend Recovery Sleep and Risk of Incident Dementia: A Prospective Cohort Study in the UK Biobank

**DOI:** 10.1002/alz70860_102436

**Published:** 2025-12-23

**Authors:** Bo Zhao, Shaojiong Zhou, Jie Chang, Aonan Li, Chaofan Geng, Tao Wei, Yiwei Zhao, Zhibin Wang, Yi Tang

**Affiliations:** ^1^ Department of Neurology & Innovation Center for Neurological Disorders, Xuanwu Hospital, Capital Medical University, National Center for Neurological Disorders, Beijing, Beijing, China; ^2^ National Center for Neurological Disorders, Xuanwu Hospital, Capital Medical University, Beijing, Beijing, China; ^3^ Innovation Center for Neurological Disorders, Department of Neurology, Xuanwu Hospital, Capital Medical University, National Center for Neurological Disorders, Beijing, China; ^4^ Department of Neurology & Innovation Center for Neurological Disorders, Xuanwu Hospital, Capital Medical University, National Center for Neurological Disorders, Beijing, Beijing, China; ^5^ Xuanwu Hospital, Capital Medical University, Beijing, Beijing, China; ^6^ Neurodegenerative Laboratory of Ministry of Education of the People's Republic of China, Beijing, Beijing, China

## Abstract

**Background:**

Sleep deprivation has been linked to a higher dementia risk, but the role of weekend recovery sleep (WRS) in reducing this risk remains unclear. The objective is to evaluate the association between WRS and dementia risk.

**Method:**

This prospective cohort study followed 88,592 dementia‐free adults aged 40–79 years from the UK Biobank, using wrist accelerometers to measure average weekday and weekend sleep durations. Incident dementia (all‐cause dementia, Alzheimer's disease [AD], vascular dementia [VaD], and nonspecific dementia) was determined using medical records. Associations were estimated using Cox proportional hazards models and restricted cubic splines (RCS).

**Result:**

Among 88,592 participants (mean [SD] age, 61.9 [7.9] years), 735 (0.83%) developed dementia, including 308 (0.35%) cases of AD, 137 (0.15%) cases of VaD, and 319 (0.36%) cases of nonspecific dementia. RCS analyses revealed optimal weekday sleep durations to minimize dementia risk: 8.38 hours for all‐cause dementia, 8.33 hours for AD, and 9.07 hours for VaD. In the suboptimal sleep group (weekday sleep less than optimal duration), longer WRS was associated with reduced risks of all‐cause dementia (HR, 0.801; 95% CI, 0.717–0.893) and VaD (HR, 0.747; 95% CI, 0.612–0.91). However, in the prolonged sleep group (weekday sleep exceeding optimal duration), longer WRS was linked to an increased VaD risk (HR, 1.425; 95% CI, 1.097–1.851).

**Conclusion:**

After insufficient weekday sleep, obtaining adequate WRS can reduce the risk of all‐cause dementia, particularly VaD. These findings underscore the importance appropriate WRS in mitigating dementia risk and improving cognitive health.

